# Modified Fabrication of Perovskite-Based Composites and Its Exploration in Printable Humidity Sensors

**DOI:** 10.3390/polym14204354

**Published:** 2022-10-16

**Authors:** Meiting Peng, Fan Zhang, Liyong Tian, Longbin You, Jiayi Wu, Nanhua Wen, Yangfan Zhang, Yancheng Wu, Feng Gan, Hui Yu, Jing Zhao, Qi Feng, Fuqin Deng, Longhui Zheng, Yingzhu Wu, Ningbo Yi

**Affiliations:** 1 School of Textile Materials and Engineering, Wuyi University, Jiangmen 529020, China; 2 Advanced Energy Storage Technology Research Center, Shenzhen Institutes of Advanced Technology, Chinese Academy of Sciences, Shenzhen 518055, China; 3 School of Applied Physics and Materials, Wuyi University, Jiangmen 529020, China; 4 Faculty of Intelligent Manufacturing, Wuyi University, Jiangmen 529020, China; 5 College of Forestry, Henan Agricultural University, Zhengzhou 450002, China

**Keywords:** perovskite, composites, humidity-sensitivity

## Abstract

Organic perovskites are promising optoelectronic semiconductor materials with photoelectric applications. It is known that the luminescence of perovskites is highly sensitive to hydron molecules due to its low moisture resistance of crystal structure, indicating its potential application on humidity-sensing. Herein, a novel perovskite-based compound (PBC) with minimal defects was developed to promote the photoluminescence performance via optimization of the drying method and precursor constitutions. Perovskite materials with good structural integrity and enhanced fluorescence performance up to four times were obtained from supercritical drying. Moreover, the hydrophilic polymer matrix, polyethylene oxide (PEO), was added to obtain a composite of perovskite/PEO (PPC), introducing enhanced humidity sensitivity and solution processibility. These perovskite/PEO composites also exhibited long-term stability and manifold cycles of sensitivity to humidity owing to perovskite encapsulation by PEO. In addition, this precursor solution of perovskite-based composites could be fancily processed by multiple methods, including printing and handwriting, which demonstrates the potential and broaden the applications in architecture decoration, logos, trademarks, and double encryption of anti-fake combined with humidity.

## 1. Introduction

In recent decades, the organic-inorganic hybrid perovskites (MAPbX_3_, where X = Cl, Br, I) have attracted significant interest with characteristics of high quantum efficiency [1,2,3], tunable optical bandgap [4,5], and highly efficient carrier mobility [6,7]. Perovskites have been intensively studied in many fields and applications owing to their excellent photoelectric efficiency and photoluminescence (PL) [8]. Especially as a promising photoelectric material, they have a wide range of applications in perovskite photovoltaic (PL), light-emitting diodes (LEDs) [9,10], low-threshold lasers [11,12], electroluminescent devices [13,14,15], photodetector [16], photocatalysts [17]. Due to their high PL quantum efficiency, perovskites were reported to as potential luminescence probes for cell imaging after overcoming the challenge of hypertoxicity by external encapsulation [18]. Therefore, the explorations in photoelectric devices and bioscience broaden the application of perovskite.

Instability under humid conditions was one of the main obstacles in the progress of hybrid perovskites and many efforts have been made to explore various ways to improve the moisture resistance of hybrid perovskites [19,20,21]. However, these studies provided a hint that hybrid perovskites could be a potential moisture-sensing material in artificial fruit waxing detection [22], breath monitoring [23], and detecting water content in herbal medicines [24]. Humidity sensors are widespread in many scientific and industrial fields, such as monitoring food quality [25], environmental conditioning [26], medical equipment [27], and material preparation [28,29]. Small organic molecules as functional layers were broadly used in humidity sensors due to their low manufacturing cost, ease and diversity of fabrication methods, and their compatibility with flexible substrates [30]. Recently, the typical capacitive humidity sensors based on naphthalene diamide derivatives, converting the molecule interaction into an electrical signal, realized a novel efficient method of humidity-sensing [31]. Based on the change in the material structure caused by the interaction with water molecules, the sensitivity of perovskite materials to humidity was achieved through mechanisms such as fluorescence [32] and electricity [20]. Haque et al. designed a detection device of MAPbI_3_ perovskite grating to obtain a high sensitivity to humidity by semiconductor patterning processing technology [33]. A good humidity-sensing material should exhibit durability for reuse in humidity sensing, periodic recovery of crystal structure, and outstanding reversibility humidity of fluorescence, which limits the pure perovskite materials applied in humidity sensors. Embedding perovskite materials into a hydrophilic polymeric matrix to form a composite is a good candidate to improve sensitivity and cycling ability, such as perovskites/metal–organic frameworks composites [34] and perovskites/covalent organic frameworks composites [35]. Additionally, the composite provides the flexibility and processability to overcome the intrinsic brittle crystal structure, such as polyurethanes [36], poly (vinyl alcohol) [37], 3-aminopropyltriethoxysilane [38], and poly(styrene-ethylene-butylene-styrene) [39] is a typical strategy to provide flexibility to perovskite materials and devices. The hydrophilic polyethylene oxide (PEO) is a good matrix combining hydrophilicity, flexibility, and processability with perovskite materials to form humidity-sensing composites, which, smooth and pinhole-free perovskite films can be achieved with small crystal domains via printing, and micro-nano imprinting as ionic insulating additives and charge blocking materials for LEDs [40,41,42,43,44,45] to enhance the photoluminescence.

Herein, a flexible perovskite-based PEO composite with promoting fluorescence in a modified fabrication process and high sensitivity to humidity was fabricated and applied to sensors through printing technology, which could be adopted for flexible and wearable opto-electric applications. Firstly, MAPbBr_3_ powder as raw material was obtained through supercritical drying promoting fluorescence efficiency. Excess MABr was then added into as-prepared perovskite into a perovskite-based compound (PBC), ensuring its stability and further enhancing its fluorescence efficiency. In addition, PEO as the matrix was composited with PBC to form a perovskite/PEO composite (PPC), which improved its processing ability and expanded its application. This PPC could achieve much higher photoluminescence than raw perovskite. PPC not only reserved the original properties of perovskite and perovskite-based compounds but was also sensitive to environmental humidity in opaqueness (apparent color) and photoluminescence, presenting potential material for sensor and encoding, relating to the reversible change of crystal structure, plane (110) and (111) reports [46]. Based on these properties, it led to the conclusion that photoluminescence was highly related to the variations in environmental humidity and was applied to design patterns sensitive to humidity in the printing method.

## 2. Experimental

### 2.1. Materials

N, N-dimethylformamide (DMF, 99.5%, aladdin, Aladdin, Shanghai, China), Dichloromethane (DCM, 99.97%, anhydrous, Aladdin, Shanghai, China), isopropanol (IPA, 99.97%, anhydrous), hydrobromic acid (HBr, 48% by weight in H_2_O, Macklin, Shanghai, China), Lead (II) Bromide (PbBr_2_, 99.99%, Macklin), Methyl ammonium bromide (MABr, 99.5%, Xi’an *p*-OLED, Xi’an, China), poly (ethylene oxide) (PEO, Mw ≈ 100,000, aladdin, Aladdin, Shanghai, China). All chemicals were used as received without any further purification and purchased from commercial resources.

### 2.2. Synthesis of MAPbBr_3_, PBC, and PPC

Briefly, 2 mmol MABr and PbBr_2_ were added into 3 mL IPA, and the solution was vigorously stirred for 45 min at room temperature. Another 5 mL HBr was added into this transparent MABr/PbBr_2_ solution, and then refrigerated centrifugation for 45 min at 12 °C. The orange MAPbBr_3_ powder was obtained after supercritical drying. Finally, the powder was stored in sealed vials for further characterization and synthesis. PBC solution was obtained by dissolving MAPbBr_3_ and MABr in DMF and DCM for 45-min stirring. PEO was then dissolved in DMF to generate a concentration of 3 mmol mL^−1^. Solutions of PEO and PBC were stirred for 45 min at 70 °C, respectively. The solutions were then mixed with the desired mass ratio and well stirred at room temperature. Finally, PEO/PBC solution was directly spin-coated to form a PPC.

### 2.3. Characterization

The absorption and photoluminescence were recorded using a UV spectrophotometer (Shimadzu, UV-2600, Tokyo, Japan) and a Fluorescence spectrophotometer (Shimadzu, RF-6000) respectively. X-ray diffraction (XRD, Rigaku/Ultima IV diffractometer, Tokyo, Japan) was adopted to analyze crystal structure. Fourier transform infrared spectroscopy (FTIR, Nicolet iS50, Waltham, MA, USA) was used to obtain the information on materials A scanning electron microscope (SEM, TESCAN Mira LMS, Brno, Czech) was adopted to characterize morphology and energy dispersive spectrometer (EDS) analysis of perovskite. The PPC pattern was drawn through the printing equipment (Prtronic DB100, Shanghai, China).

## 3. Results and Discussion

### 3.1. Preparation Process for Perovskite

**Figure 1:**
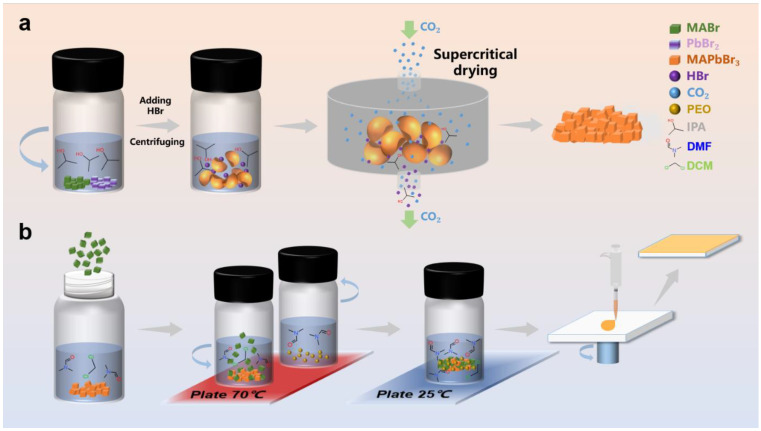
Preparation of MAPbBr_3_, PBC, and PPC. (**a**) Schematic illustration of the preparation of hybrid perovskite by supercritical drying. (**b**) Schematic illustration of the facile synthesis of halide-rich hybrid perovskite nanocomposite films. The blend solution was made from MAPbBr_3_, MABr, and PEO powder, then directly spin-coated into a bromine-rich nanocomposite film.

The Br-perovskite studied in this work was prepared via antisolvent and supercritical drying as shown in Figure 1a. Two components (MABr and PbBr_2_) were added into isopropanol (IPA) at a stoichiometric ratio to form a precursor solution. Hydrobromic acid was then added to the precursor solution to obtain the sediment, perovskite crystals. According to a previous report, an excess molar ratio of MABr could lead to high-quality perovskite for materials and devices [47,48]. Herein, MABr was added into the solution as prepared from previous MAPbBr_3_ powder and recrystallized by anti-solvent following supercritical drying, same as the fabrication process to obtain perovskite-based compounds in Figure 1b. The performance and fluorescence brightness of the PBC films were indeed much better than those of spin coated with pure MAPbBr_3_ solution (Figure S1a). However, perovskite and it is compound both process a brittle crystal structure, which restricts operation method, device morphology, and application in some flexible technologies. Methods in preparing perovskite/polymer composites were thus studied to overcome the crystal defects, in which the polymer served as a matrix. Comprehensive studies were carried out in terms of the selection of polymer matrix, compatibility, and properties of composites as a whole. The drying process of the perovskite solution could lead to composite surface defects due to its complex hydrodynamics [49,50,51]. In previous studies, PEO was suggested as a potential matrix to improve the film and device quality in perovskite-based composites [52,53,54]. Figure 1b was a schematic illustration of the facile synthesis of PPC films.

### 3.2. Structures and Optical Properties of Perovskite

**Figure 2:**
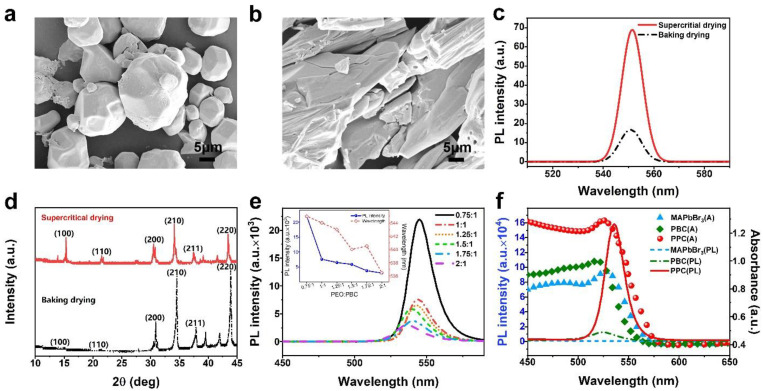
PL characterization of perovskite. SEM images of (**a**) supercritical drying and (**b**) baking drying at 5 μm. (**c**) The photoluminescence image of MAPbBr_3_ under both baking and supercritical drying methods, with an excitation wavelength of 365 nm. (**d**) XRD spectra related to different dry methods. (**e**) The fluorescence image of PPC films with different mass ratios of PEO/PBC in RH 10%. Inset: The image of PPC of variation of peak intensity and wavelength with PEO/PBC at different mass ratios. (**f**) The photoluminescence (the lines) spectra and absorption (the symbols) at the regions labeled MAPbBr_3_ (blue line and blue triangle), PBC (green line and green rhomb), and PPC (red line and red sphere).

The crystal and structural integrity are key factors in the properties of perovskite, depending on the drying condition. Supercritical drying, an effective method for removing surface solvent on crystals and retaining the full perovskite structural integrity, was adopted in this work to eliminate the solvent [55]. SEM images showed that perovskite obtained from supercritical drying (Figure 2a) was more likely to be a single-crystal structure than that from baking (Figure 2b) in the same fabrication process. This was demonstrated by the photoluminescence spectra in Figure 2c and XRD analysis in Figure 2d. The perovskite obtained from supercritical drying possessed a higher intensity of photoluminescence compared to that from the conventional drying method in Figure 2c. Figure 2d demonstrated that the typical crystal plane (110), which is related to perovskite, could form easily during supercritical drying. Figure S2 exhibited EDS analysis of perovskite forming from both supercritical drying and baking methods. The perovskite from supercritical drying was demonstrated to be a good functional candidate for the development of perovskite composites. PEO, a hydrophilic material absorbing moisture due to its hydrogen bonding, is widely used as woven, biology, and engineering materials [56,57,58]. Perovskite and its compounds could be dissociated in water or other polar solvents [59,60]. Due to the environmental hydrone vapor absorbed by PEO, an increased content of PEO could dramatically reduce the PL intensity of PPC under the relative humidity (RH) of 50% (Figure 2e). It suggested that PPC materials could be a potential material for humidity-sensing. The ratio of 0.75:1 was adopted in this work, which possessed a remarkable fluorescence character and formed a smooth film (Figure S1c). PPC could maintain the properties and structural integrity of PBC embedded in PEO, shown in XRD, EDS, and FTIR spectra. As the structure investigation in XRD (Figure S1b), the PPC could restore characteristic planes of perovskite and PBC, (100), (110), and (200). In Figure S2c,d, the Br/Pb ratio in PPC could keep around 90.7/9.3, which agreed well with the ratio of 88.07/11.93 in PBC. In the FTIR analysis of PBC and PPC (Figure S3), the peak around 1570 cm^−1^ was assigned to the stretching vibrations of C-N in PBC and the peak at 1112 cm^−1^ was assigned to C-O-C in PEO, which demonstrated the two constitutions of PPC composites [61,62]. In addition, the photoluminescence and UV-vis spectra from PBC to PPC were shown in Figure 2f, demonstrating that the addition of PEO could restore the properties of PBC and enhance the optical characteristic due to DDA theory [54].

### 3.3. Humidity Sensitivity of PPC Materials

**Figure 3:**
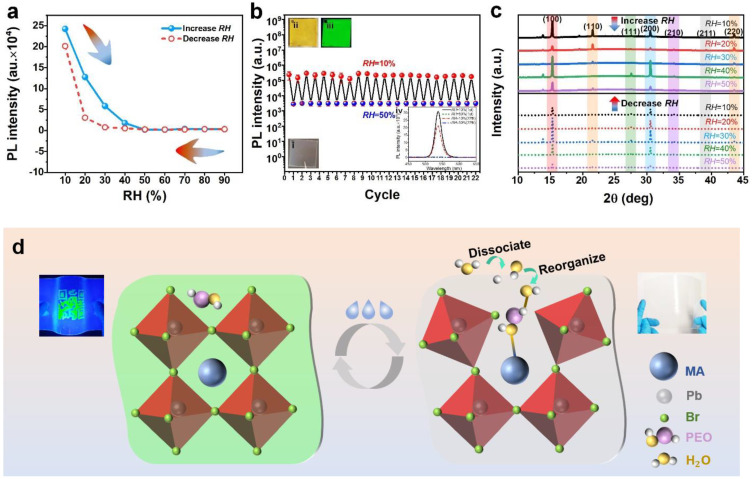
PL sensitivity and mechanism of PPC material. (**a**) PL spectra of PPC film under a single humidity cycle (the RH of 10%~90%). (**b**) The change of PL intensity with 22 cycles in reversible humidity (Note: a cycle of 10 samples). Inset: (i) Natural light photos under 50% humidity, (ii) Natural light and (iii) UV photos under 10% humidity, (iv) The PL spectra of initial PPC film and that after several humidity cycles. (**c**) XRD of PPC film in increase and decrease of humidity under the RH of 10%~50%, respectively. (**d**) Schematic illustration of the mechanism of fluorescence reversibility of PPC materials.

As a former exploration and analysis of PPC materials, it could be a good candidate for a humidity sensor. Humidity-dependence of PL spectra of PPC was investigated by varying RH from 10% to 90%, indicating that PL efficiency and intensity of PPC were sensitive to environmental humidity in Figure 3a. PL intensity of PPC decreased dramatically with the increase of RH and recovered after the RH decrease. The related PL spectra of PPC samples in different humidity environments were shown in Figure S4. It concluded that the PL of PPC could show a high response to the relative humidity in the range of 10–50%. To ensure the authenticity of the results to humidity sensitivity is not by chance, the PL intensity of 10 samples was investigated for statistics of cycling stability under two humidity states (10% and 50%) in Figure 3b. It could achieve the 22 cycles of PL intensity sensing to RH in this work, indicating the sustainable humidity sensitivity and stability of PPC in a moisture environment. It was noted that the PPC materials became non-colored, semi-transparent, and non-PL in the 50% humidity condition (inset i and iv of Figure 3b). After being placed in a low (10%) humidity environment, color and PL properties recovered immediately as shown in inset ii and iii of Figure 3b, suggesting the potential application of a sensitive indicator for humidity. The morphology of the PPC sample after humidity cycles was shown in the SEM images (Figure S5). This PL sensitivity of PPC materials to RH relied on the crystal structure. Therefore, the structural variation of PPC in different humidity conditions was revealed in Figure 3c and Figure S6. According to the XRD analysis, the plane (110) of PPC was strongly correlated with humidity, which can affect color and PL properties. The PL sensitivity to RH could recover after environmental humidity is reduced (inset iv of Figure 3b), corresponding to the recovery of crystal structure in Figure 3c. Therefore, the sensing mechanism is that the interaction between hydrone absorbed by PEO and N-H of MA^+^ group results in the collapse of typical crystal structure with humidity increase, leading to non-coloration and reduced PL intensity (Figure 3d). However, this could recover and could lead to the reconstruction of its crystal structure after the decrease of RH because there is no absence of precursor in perovskite encapsulated by PEO, which leads to the cycling ability of humidity-sensing. Based on this mechanism, the PPC illustrates the potential applicability of humidity sensors.

### 3.4. Printable Sensing Devices from PPC

**Figure 4:**
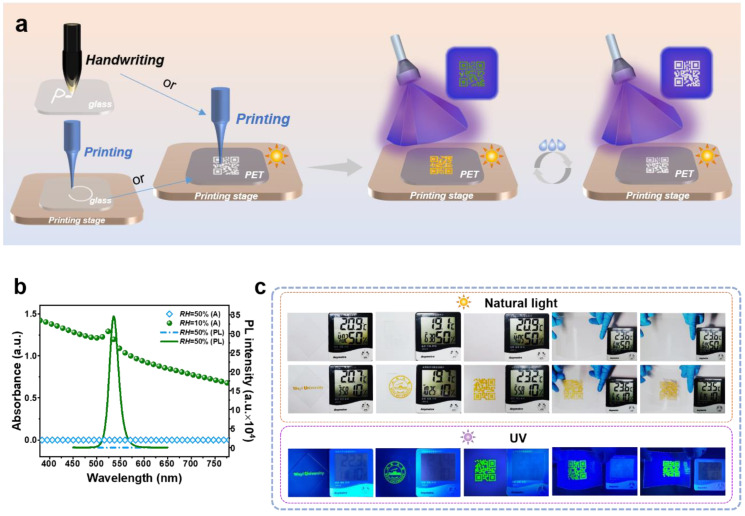
Application of PPC in dual sensing of temperature and humidity. (**a**) Schematic drawing of reversible fluorescent patterns on glass substrates and PET substrates by handwriting or printing. (**b**) UV-vis and PL spectra at different RH. (**c**) Humidity-sensitive words and patterns from PPC materials were designed via printing and writing on glass slides and flexible PET substrates.

Due to the constitution of PEO and PBC, PPC could be dissolved in many solvents, including DMF and DMSO. It behaves as a liquid state of polymer resin, which could be applied in handwriting, printing, molding, or other processing methods. After evaporation of the solvent, PPC can form many shapes and structures, also retain its intrinsic sensitive properties. Printing is a promising method widely used in fields of materials, engineering, biology, and devices, unlimited by shapes, morphologies, and forms. Herein, PPC materials were reformed on glass slides and flexible PET substrates by printing and using an ink brush (Figure 4a). In addition, the optical performance of the humidity sensitivity of PPC applications was described in Figure 4b–d. According to Figure 4b, there is little absorbance in a visible light band in high RH. Furthermore, there is no typical fluorescence peak existence in PL spectra under this condition. When the humidity decreased, the absorbance was promoted, and the PL peak appeared immediately. Based on this characteristic, several humidity-sensitive patterns from PPC materials were designed via printing and writing on glass slides or flexible PET substrates in Figure 4c. The printed patterns could be hidden under high environmental humidity (RH 50%) and appeared under low environmental humidity (RH 10%). This humidity sensitivity demonstrated that PPC could also be applied to encrypted fluorescent anti-counterfeiting QR codes. The flexible humidity sensors could be designed and obtained on the flexible substrates (Figure 4c), which could be used as a humidity indicator on the package of moisture-proof products.

## 4. Conclusions

In summary, the perovskite-based compound material was obtained by antisolvent and supercritical drying, presenting enhanced performance in PL intensity. From the analyses of crystal structure, defects in perovskite crystal could be effectively eliminated by adopting excessive MABr. Additionally, embedding perovskite-based compounds into a polymer matrix (PEO) to form a perovskite/PEO composite, could preserve the properties of the perovskite-based compound and promote processability in flexible devices. In addition, it is interesting that the PL intensity of this nanocomposite and its devices exhibited high sensitivity to humidity. Based on sensitivity and solution-processability, various patterns could be designed and obtained through handwriting and printing, which demonstrated that PPC materials could be broadly applied in architecture decoration, logos, trademarks, and double encryption of anti-fake corresponding to humidity.

## Data Availability

Not applicable.

## Supplementary Materials

The following supporting information can be downloaded at: https://www.mdpi.com/article/10.3390/polym14204354/s1, Figure S1: (a) The image of MAPbBr_3_ and PPC film under natural light and UV light. (b) XRD image of PEO/MAPbBr3/PBC/PPC. (c) The image of PEO: PBC films in different mass ratio under natural light and UV light at RH 10%, Figure S2: EDS images of every phase. MAPbBr_3_ in (a) supercritical drying and (b) bakeing drying, (c) PBC film, (d) PPC film. (e) PPC film after experiencing RH, Figure S3: FTIR spectra in transmittance scales for MAPbBr3, PBC, and PPC films, Figure S4: PL spectra of PPC in (a) increase and (b) decrease of humidity under the RH of 10%~50%, respectively, Figure S5: SEM images of PPC film before and after experiencing RH cycles, Figure S6: XRD of composite film of the initial humidity of 10% and the final humidity of 50%.
