# Supplementary material for: Modified Fabrication of Perovskite-Based Composites and Its Exploration in Printable Humidity Sensors
Source: Polymers (Basel). 2022 Oct 16;14(20):4354. doi: 10.3390/polym14204354 (PMC9606918; doi:10.3390/polym14204354)
Supplement: Supplementary file 1 [file polymers-14-04354-s001.zip › polymers-1976453-supplementary.pdf]

Supporting Information

Modified Fabrication of Perovskite-Based Composites and Its Exploration in Printable Humidity Sensors

Meiting Peng [1,†], Fan Zhang [2,†], Liyong Tian [1,†], Longbin You [1], Jiayi Wu [1], Nanhua Wen [1], Yangfan Zhang [1],

Yancheng Wu [1], Feng Gan [1], Hui Yu [1], Jing Zhao [1], Qi Feng [3], Fuqin Deng [4], Longhui Zheng [5], Yingzhu Wu [1,] and Ningbo [Yi 1,*]*

[1]School of Textile Materials and Engineering, Wuyi University, Jiangmen 529020, China

[2]Advanced Energy Storage Technology Research Center, Shenzhen Institutes of Advanced Technology, Chinese Academy of Sciences, Shenzhen 518055, China

[3]School of Applied Physics and Materials, Wuyi University, Jiangmen 529020, China

[4]Faculty of Intelligent Manufacturing, Wuyi University, Jiangmen 529020, China

[5]College of Forestry, Henan Agricultural University, Zhengzhou 450002, China

*Correspondence: wyz@wyu.edu.cn (Y.W.); yiningbo@wyu.edu.cn (N.Y.)

† These authors contributed equally to this work.

Supplementary figures

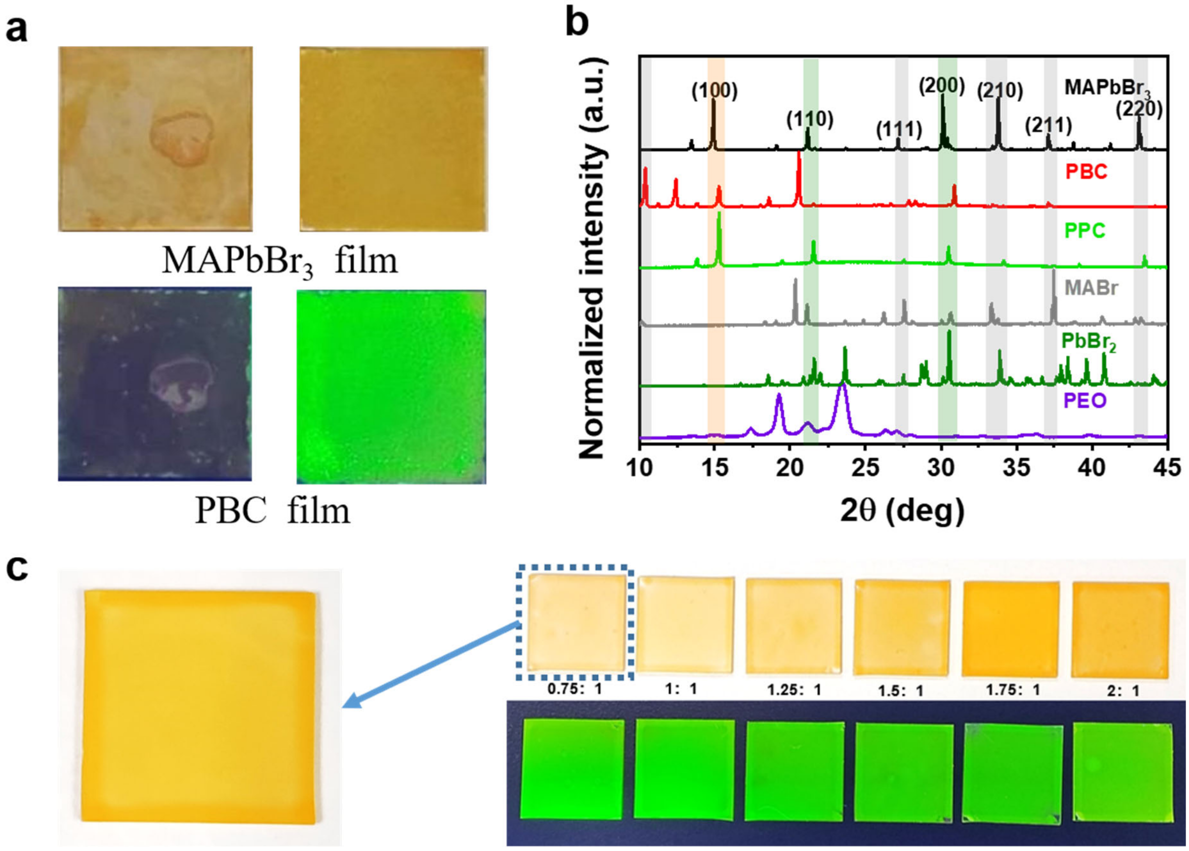

Figure S1. (a) The image of MAPbBr3 and PPC film under natural light and UV light . (b) XRD image of PEO/MAPbBr3/PBC/PPC. (c) The image of PEO:PBC films in different mass ratio under natural light and UV light at RH 10%.

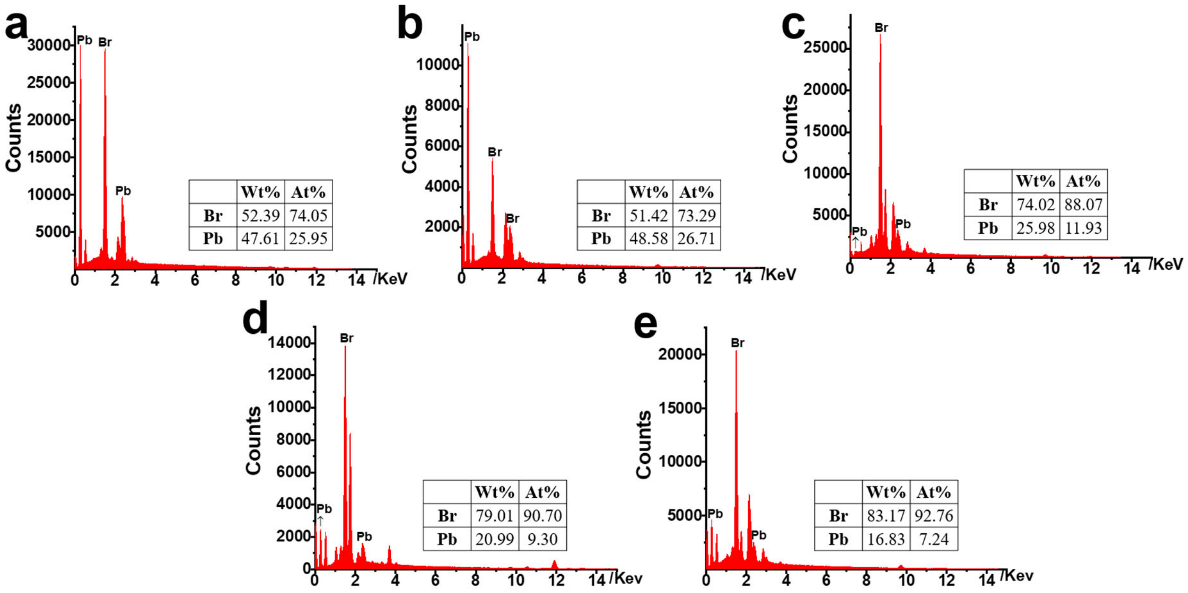

Figure S2. EDS images of every phase. $MAPbBr_3$ in (a) supercritical drying and (b) bakeing drying. (c) PBC film. (d) PPC film. (e) PPC film after experiencing RH.

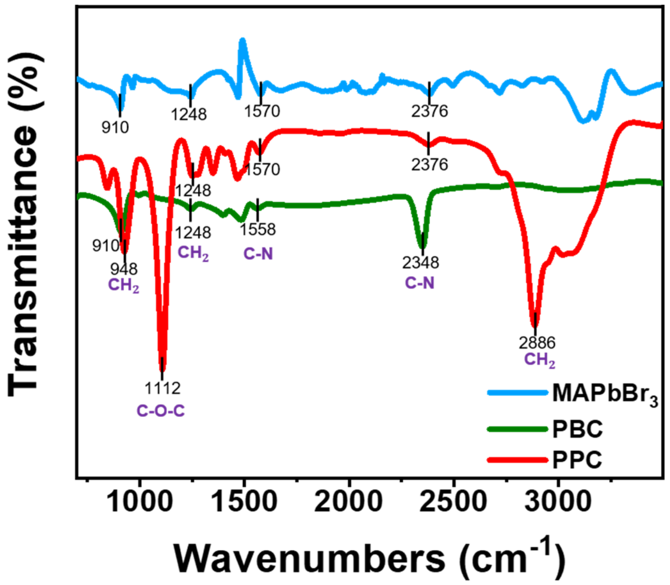

Figure S3. FTIR spectra in transmittance scales for $MAPbBr_3$, PBC, and PPC films.

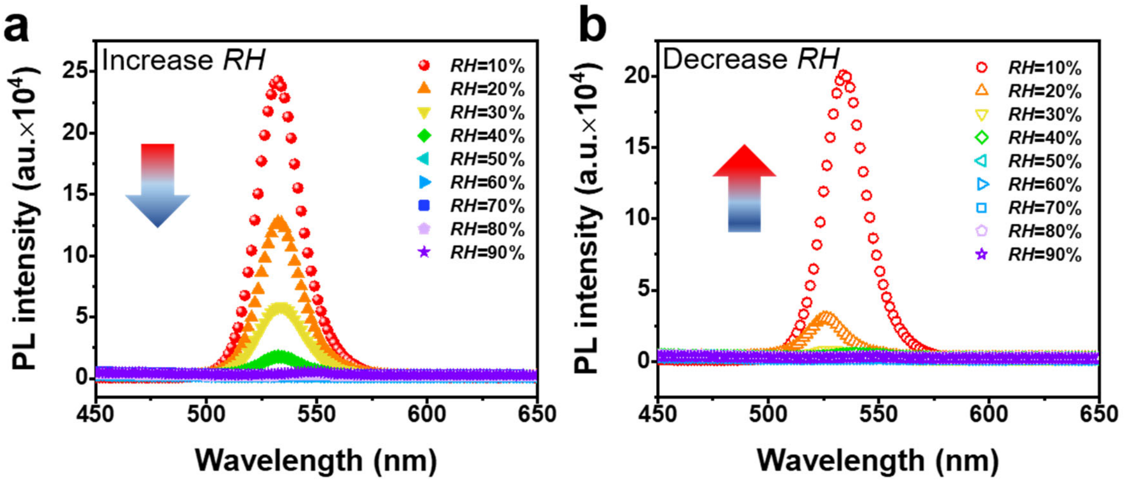

Figure S4. PL spectra of PPC in (a) increase and (b) decrease of humidity under the RH of 10%~50%, respectively.

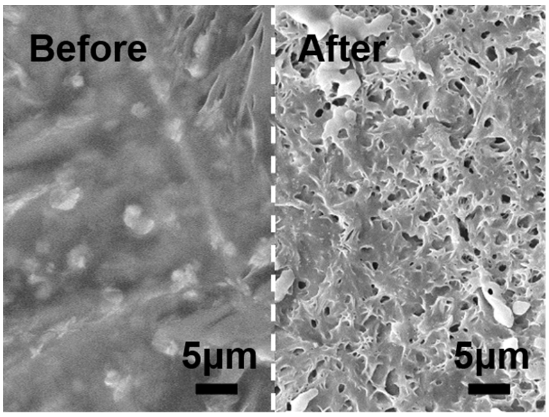

Figure S5. SEM images of PPC film before and after experiencing RH cycles.

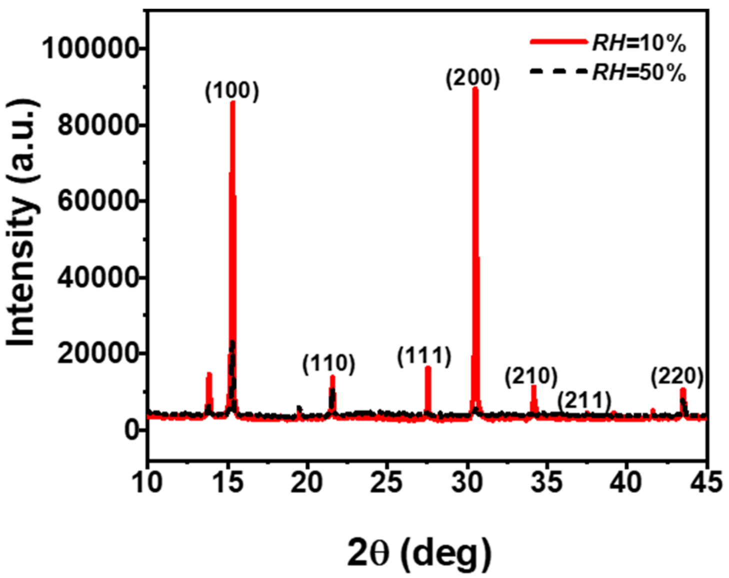

Figure S6. XRD of composite film of the initial humidity of 10% and the final humidity of 50%.
